# Topical Delivery of Aceclofenac: Challenges and Promises of Novel Drug Delivery Systems

**DOI:** 10.1155/2014/406731

**Published:** 2014-06-18

**Authors:** Kaisar Raza, Manish Kumar, Pramod Kumar, Ruchi Malik, Gajanand Sharma, Manmeet Kaur, O. P. Katare

**Affiliations:** ^1^Department of Pharmacy, School of Chemical Sciences & Pharmacy, Central University of Rajasthan, Bandarsindri, Ajmer District, Rajasthan 305 801, India; ^2^Division of Pharmaceutics, University Institute of Pharmaceutical Sciences, Panjab University, Chandigarh 160 014, India

## Abstract

Osteoarthritis (OA), a common musculoskeletal disorder, is projected to affect about 60 million people of total world population by 2020. The associated pain and disability impair the quality of life and also pose economic burden to the patient. Nonsteroidal anti-inflammatory drugs (NSAIDs) are widely prescribed in OA, while diclofenac is the most prescribed one. Oral NSAIDs are not very patient friendly, as they cause various gastrointestinal adverse effects like bleeding, ulceration, and perforation. To enhance the tolerability of diclofenac and decrease the common side effects, aceclofenac (ACE) was developed by its chemical modification. As expected, ACE is more well-tolerated than diclofenac and possesses superior efficacy but is not completely devoid of the NSAID-tagged side effects. A series of chemical modifications of already planned drug is unjustified as it consumes quanta of time, efforts, and money, and this approach will also pose stringent regulatory challenges. Therefore, it is justified to deliver ACE employing tools of drug delivery and nanotechnology to refine its safety profile. The present review highlights the constraints related to the topical delivery of ACE and the various attempts made so far for the safe and effective topical delivery employing the novel materials and methods.

## 1. Introduction

Osteoarthritis (OA) is defined by WHO as a condition characterized by focal areas of loss of articular cartilage within the synovial joints, associated with hypertrophy of the bone (osteophytes and subchondral bone sclerosis) and thickening of the capsule. The disease most commonly affects the middle-aged and elderly patients, with estimated worldwide prevalence of 9.6% for men and 18.0% for women aged at least 60 years, although younger people are also on the disease target as a result of injury or overuse [1]. OA is characterized by joint pain, tenderness, stiffness, crepitus, and local inflammation. The most commonly affected joints are those of hand followed by the knee joints, and the disease usually impairs the mobility and physical activity due to increasing levels of pain, thus posing a detrimental impact on a patients' quality of life and their ability to perform normal daily activities [1–4].

Nonsteroidal anti-inflammatory drugs (NSAIDs) are the most commonly prescribed drugs for the treatment of OA. NSAIDs are definitely better than placebo and are enjoying the status of popular “over the counter” medicines amongst the health professionals and the patients [5]. Diclofenac, a US-FDA approved drug in 1988, is the most commonly prescribed NSAID for the treatment of OA-related pain. The efficacy of diclofenac is still believed to be unmatchable as it is as effective as the newer approved pain relief medications for OA and continues to be a benchmark pharmacological treatment option for OA to the physician [6]. Despite several advantages, diclofenac is also associated with the NSAID-category side effects like gastrointestinal (GI) adverse effects including bleeding, ulceration, and perforation of the stomach, small intestine, or large intestine, which can be fatal too. These drawbacks of a timely tested drug always motivated the medicinal chemists to develop a new/modified NSAID with enhanced safety and comparable efficacy. This driving force resulted in the development of ACE, that is, a derivatized diclofenac developed by Grau et al. in 1991 to improve its gastrointestinal tolerability [7, 8].

ACE offered a relatively better gastric tolerance* vis-à-vis *the other NSAIDs including diclofenac [9]. The incidence of gastric ulcerogenicity of ACE has been reported to be significantly lower than that of the other frequently prescribed NSAIDs, for instance, 2-folds lesser than naproxen, 4-folds lesser than diclofenac, and 7-folds lesser than indomethacin [10]. ACE is also expected to provide economic benefits owing to its better tolerability and marked efficacy [11]. On pharmacokinetic fronts, ACE is well absorbed from gastrointestinal tract and circulates mainly as unchanged drug, while the food presence rarely alters its pharmacokinetic properties [10]. Model independent pharmacokinetic parameters like *C*_max⁡_, *V*_*d*_, and half-life as well as the absorption of ACE are not affected by escalating age and, therefore, dose manipulations are not generally advocated in the elderly patients [11]. Though reported to be well-tolerated, a few incidences of rare hypersensitivity reactions after oral intake of ACE are reported including hypersensitivity vasculitis [12], photoallergic contact dermatitis [13], exudative erythema multiforme [14], anaphylactic reaction [15], and acute tubulointerstitial nephritis [16]. Also, two NSAIDs with similar chemical structure with ACE, namely, alclofenac and fenclofenac, have been associated with higher incidences of rashes and, subsequently, withdrawn in late 1970s and 1980s, respectively [12, 15].

## 2. Mechanism of Action of Aceclofenac

The mode of action of ACE is mainly based on the inhibition of synthesis of prostaglandins (PG). ACE inhibits the cyclooxygenase (Cox) enzyme, which is involved in the synthesis of PG [17].* In vitro* data in unstimulated bovine aortic coronary endothelial cells indicated the selectivity for Cox-2 by ACE more than Cox-1 [18]. ACE also inhibits the synthesis of the inflammatory cytokines, interleukins, and tumor necrosis factors. Also, effect of ACE on the cell adhesion molecules from the neutrophils has also been proposed [19].

**Figure 1:**
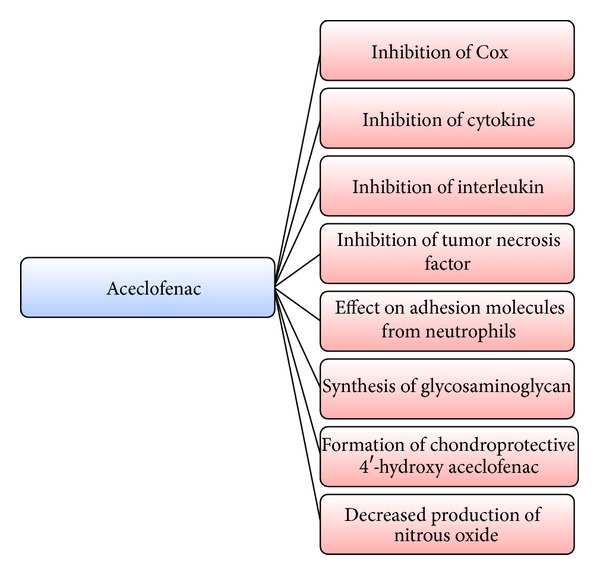
Targets of ACE resulting in decrease of pain and inflammation.

Its interleukin-1 (IL-1) inhibition activity may be linked to its stimulatory effects on cartilage matrix by release of glycosaminoglycan [20] and a chondroprotective agent, 4′-hydroxyacelofenac [21, 22]. The decreased production of nitrous oxide in human articular chondrocytes is also linked to its anti-inflammatory activity [23]. As 4′-hydroxy aceclofenac participates in chondroprotection by interfering with IL-1-mediated production of promatrix metalloproteinase-1 and metalloproteinase-3 and the release of proteoglycans from chondrocytes, ACE is classified as a novel NSAID. It simultaneously downregulates the production of promatrix metalloproteinases as well as prostaglandin E2 in osteoarthritis and/or rheumatoid arthritis [20]. Surprisingly, ACE is not involved in the tendon cell proliferation unlike indomethacin and naproxen and can be safely prescribed for the treatment of pain after tendon injury and surgery [24]. In patients with OA of the knee, ACE decreases pain resulting in reduction of disease severity and improves the functional capacity of the knee. It reduces joint inflammation, pain intensity, and the duration of morning stiffness in patients with rheumatoid arthritis [25, 26].  Figure 1 shows the various targets affected by ACE which result in overall reduction of pain and inflammation.

## 3. Need of Nonoral Delivery

ACE is frequently prescribed by oral route for the management of OA [25, 26]. Though efficacious and relatively safer, chronic oral intake of ACE can result in NSAID-specific toxicity. The oral delivery of ACE is challenging and not always justified approach. Chronic oral ACE administration leads to unfavorable effects especially on the gastric mucosa due to PG inhibition. These side effects can be precipitated as simple infirmities like dyspepsia, moderate problems like peptic ulcers, and severe concerns like gastrointestinal haemorrhage [27]. Despite this, ACE has the potential to cause local irritation and gastrointestinal mucosal lesions due to its acidic character [28].

**Figure 2:**
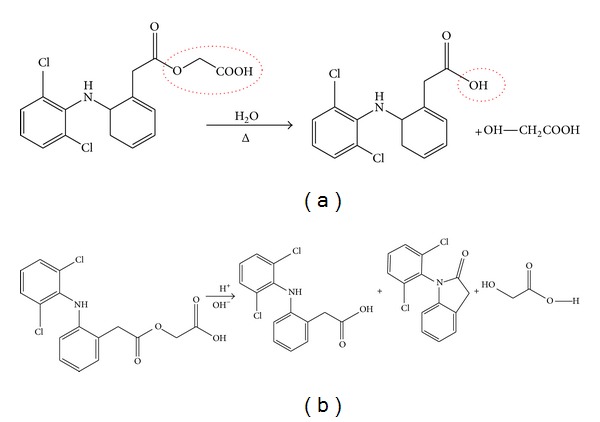
(a) Hydrolysis of ester linkage of ACE to give diclofenac and glycolic acid. (b) Hydrolysis of ACE to give diclofenac, 1-(2,6-Dichlorophenyl)-2-indolinone and glycolic acid.

ACE belongs to BCS Class II and possesses poor aqueous solubility of the order of 60 *μ*g/mL [29]. It is well established that the bioavailability of poorly water-soluble drugs is a dissolution limited step and is a critical parameter as very low solubility in biological fluids generally results in poor bioavailability after oral administration [30]. Apart from therapeutic challenge of side effects, physicochemical challenges of low aqueous solubility, and higher log *P*, ACE also pretenses a chemical challenge of instability in acidic, alkaline, and neutral media as well as in light. Substantial degradation of ACE has been reported in various media and light conditions by Bhinge et al. [31]. However, the major degraded product of ACE is diclofenac as shown in Figures 2(a) and 2(b) [31–34].

For the ease of application, enhanced stability, decreased side effects, enhanced patient compliance, and the ease of discontinuation on desire, there have been a lot of efforts to deliver ACE by topical route [35–37]. This route of delivery is frequently proposed by the formulation scientists to avoid the gastric side effects of various drugs. Apart from this, the chances of drug-drug interactions will also be minimized by topical administration of ACE. It is also reported that topical NSAIDs require overall less active drug* vis-à-vis *the dosage of systemic NSAIDs. Therefore, topical therapy is not only promising on the safety and efficacy fronts but also on the economic fronts too [38]. The various advantages of topical delivery are enlisted in Table 1.

## 4. Challenges in Topical Delivery

**Figure 3:**
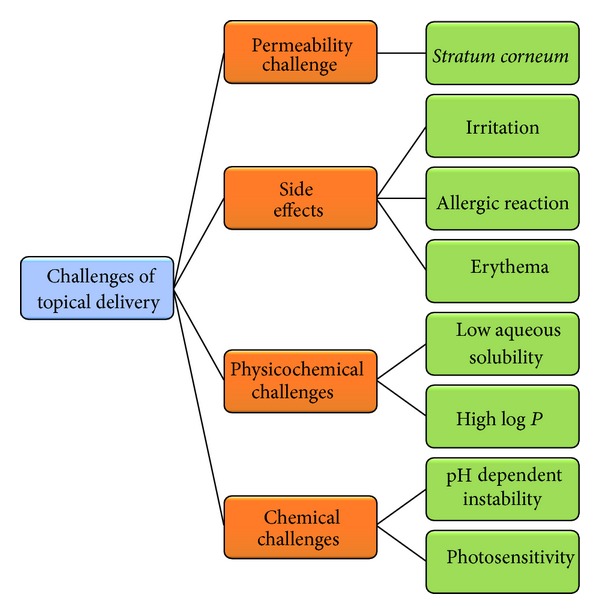
Various challenges posed by ACE to formulation scientists [35–40].

The greatest challenge for dermal penetration is the tough horny layer, that is,* stratum corneum* (SC), the uppermost layer of the skin, which is the rate limiting step for epidermal drug transport [39]. The physicochemical factors of drug like log *P*, pK_*a*_, solubility, and molecular mass also play an important role in the selection of components for the topical delivery vehicle [36, 40]. For acidic and unstable drugs like ACE, special consideration has to be made on the excipient selection for topical vehicle which will not only mask the irritation potential of ACE due to acidic group but also provide* milieu *for effective topical delivery and maintenance of chemical integrity [35, 38].  Figure 3 highlights the various challenges offered by ACE while developing an ideal topical formulation.

## 5. Novel Drug Delivery Systems

**Figure 4:**
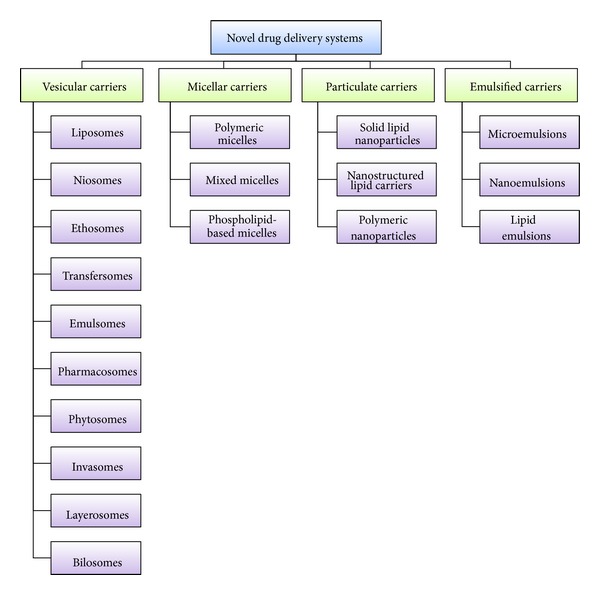
Various drug delivery carriers studied as a part of NDDS [35, 36, 40–54].

Most of the drug delivery systems, called novel drug delivery systems (NDDS), are not now novel as exponential research has been made by scientists in this domain globally but are promising drug delivery carriers. NDDS include various drug delivery systems as shown in Figure 4 [35, 36, 40–54].

**Figure 5:**
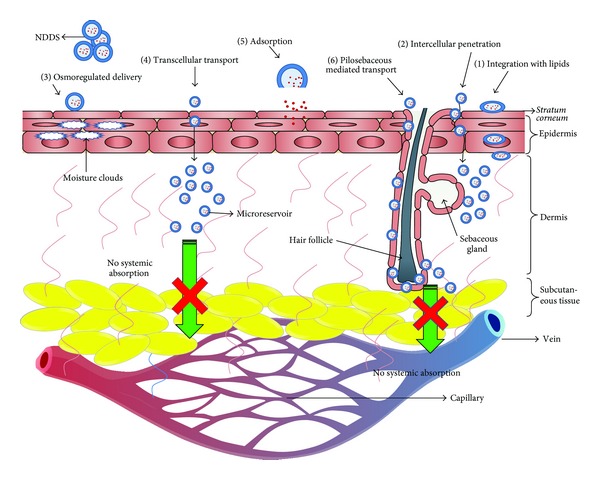
Various mechanisms of penetration of drug-loaded NDDS across skin [35, 56–58].

The conception of the concept of NDDS can be linked to the Nobel Laureate, Sir Paul Ehlrich (in 1905), who envisioned the drug molecule as “magic bullets” which can hit the desired site only to exhibit the effect. Somehow, the concept of “magic bullets” transformed to “magic guns,” that is, NDDS [35, 55]. As shown in Figure 5, these carriers interact with skin components and effectively deliver the loaded drug to the various layers of skin [35]. Depending on their compositional attributes, these carriers can deliver the drug to various layers of skin by one or many processes, as highlighted in Figure 5. (1) Carriers composed of biocompatible and biosimilar excipients like liposomes and microemulsions can integrate with the lipids of the biological membranes. (2) Smaller carriers like lipid nanoparticles (SLNs/NLCs), micro- and nanoemulsions, and flexible carriers like ethosomes and flexible membrane vesicles (FMVs) can pass through the intercellular spaces of the skin cells and can deliver the drug. (3) The moisture cloud beneath the SC can trigger osmoregulated delivery for elastic vesicles like FMVs and ethosomes. (4) Small lipid-based carriers like SLNs/NLCs also penetrate the skin* via *transcellular pathway, that is, through the keratinocytes. (5) The large-sized population of the colloidal carriers get adsorbed to the SC and release the drug by diffusion. (6) The transappendageal route involves the passage through the hair follicles and sweat and sebaceous glands and is nowadays regarded as the one of the major routes of drug transport by NDDS [35, 56–58].

These carriers are promising and many products have been approved by US-FDA based on these carriers [35].  Table 2 highlights the various advantages of these carriers [35, 52].

In recent past, there has been an exponential increase in the research pertaining to delivery of variety of bioactives employing these promising carriers. This has drifted many successful products based on these carriers to the market. Presently, there are approximately 500 carrier-based drug products catalogued and dozens of them have been approved by various federal agencies like US-FDA, DCGI, and EMEA. The projected market of such products is estimated to grow up to $3.1 trillion by the year 2015 [59]. A few successful examples, for instance, include Ambisome (liposomal amphotericin B), Lipusu (liposomal paclitaxel), Psorisome (liposomal dithranol), Neoral (cyclosporine microemulsion), Fungisome (liposomal amphotericin B), and a large number of cosmeceuticals based on solid lipid nanoparticles and nanostructured lipid carriers [35, 60–63].

## 6. Attempt for Topical Delivery of Aceclofenac Employing NDDS

Considering the benefits of topical delivery* vis-à-vis *the conventional oral administration, several attempts have been made for better topical delivery of ACE employing NDDS.

**Figure 6:**
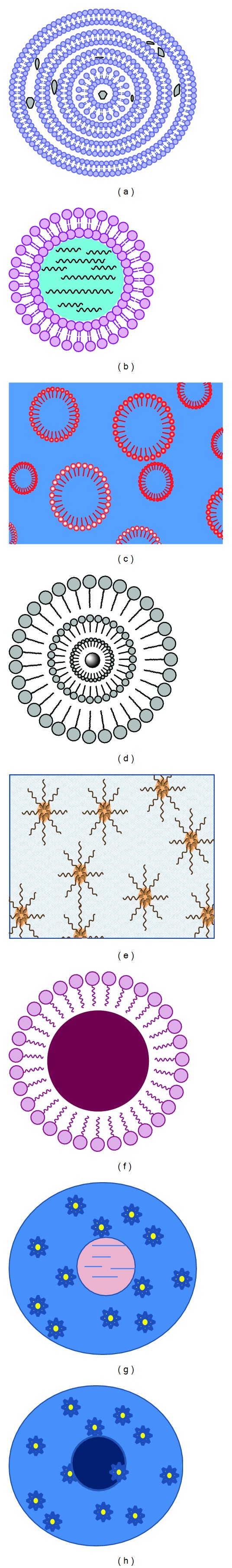
Pictorial representation of various NDDS employed for the topical delivery of aceclofenac: (a) liposomes, (b) ethosomes, (c) micro- and nanoemulsions, (d) niosomes, (e) organogels, (f) lipospheres, (g) NLCs, and (h) SLNs.

Table 3 shows the various efforts made in this required domain and a brief account of these efforts has been presented in the subsequent section. For the better understanding of the NDDS, illustrations of the discussed drug delivery carriers have been presented in Figure 6.

### 6.1. Liposome-Mediated Delivery

Liposomes are unilamellar or multilamellar vesicular structures composed of phospholipid molecules assembled into bilayers and have been extensively investigated for their potential application in pharmaceutics including drug delivery [39, 64], drug targeting [65], and controlled drug release [66]. These vesicles contain aqueous and lipidic compartment(s), as shown in Figure 6(a) and, hence, can load variety of drugs. Despite all these, liposomes are composed of biocompatible phospholipids, which are the natural components of biological membranes, and therefore they belong to the widely studied categories of NDDS [35]. Nasr et al. developed multilamellar ACE-loaded liposomes and reported significant sustained anti-inflammatory activity assessed on carrageenan-induced rat paw oedema* vis-à-vis *the marketed product. However, liposomes widely explored for other drugs seem to be unexplored for the delivery of ACE [67].

### 6.2. Ethosomes-Mediated Delivery

Ethosomes are phospholipid-based vesicles like liposomes but contain higher levels of alcohol. It has also been demonstrated that ethosomal components can reach deeper layers of the skin and can also enter the systemic circulation [68]. These carriers are generally devoid of cholesterol and offer higher skin permeation flux and drug transport [35, 68]. As portrayed in Figure 6(b), these carriers contain phospholipid bilayer enclosed hydroalcoholic chambers and can be advantageous to load alcohol-soluble drugs. Lewis and Dave developed ACE-loaded ethosomes comprising two alcohols, namely, ethanol and propylene glycol. The developed system offered enhancement in permeation more than the marketed product and was also stated to possess substantial stability [69]. Dave et al. employed higher amounts of isopropyl alcohol (40%) to load ACE in the ethosomal vesicles and reported enhanced transdermal permeation flux for the studied system [70]. Barupal et al. employed both ethanol and propylene glycol to formulate ACE-loaded ethosomes and a slight improvement in permeation was achieved [71]. Garg et al. reported more enhanced anti-inflammatory efficacy of ethosome-entrapped ACE than the marketed product [72].

### 6.3. Microemulsion and Nanoemulsion-Mediated Delivery

Microemulsions are isotropic, transparent, thermodynamically stable mixtures of water, oil, and surfactant [50, 51] whereas nanoemulsions are thermodynamically stable transparent/translucent dispersions of oil and water stabilized by an interfacial film of surfactant and cosurfactant molecules [73]. These emulsified systems have the potential to enhance the dermal permeation of lipophilic as well as hydrophilic drugs and offer higher drug loading. Essentially, these systems contain the oil globules emulsified by surfactant(s) formed micelles, as shown in Figure 6(c). Yang et al. developed ACE-loaded microemulsion and reported enhanced skin permeability and efficacy of ACE. The anti-inflammatory efficacy of ACE-microemulsion was evaluated on healthy human volunteers with experimental delayed onset muscle soreness (DOMS) and found superior to the ACE cream formulation. These studies provide the first evidence of ACE permeation in human subjects [74]. Lee et al. developed microemulsions with terpenes as the penetration enhancers, whereas limonene is the best one, and reported manyfold skin permeability more than that of ethanolic ACE formulation [75]. Shah et al. developed microemulsion employing isopropyl myristate as the oil and carried out various characterization studies for microemulsions [76]. Shakeel et al. formulated ACE-loaded nanoemulsions and reported promising potential for transdermal drug delivery. Enhanced transdermal ACE permeation was observed with the nanoemulsion* vis-à*-*vis* the conventional and niosomal gels. The anti-inflammatory efficacy was found to be superior to that of ACE gel formulations assessed on carrageenan-induced hind paw edema model. The formulation was also found to be nonirritant when tested on the skin of albino mice [77]. A detailed description of topical ACE-nanoemulsion with enhanced skin permeability and anti-inflammatory efficacy has been presented recently by Dasgupta et al. [78].

### 6.4. Niosome-Based Delivery

Niosomes are the unilamellar or multilamellar vesicles composed of nonionic surfactants and are capable of entrapping hydrophilic and hydrophobic drugs [79]. Niosomes are projected as more promising drug carriers than liposomes as they possess greater stability and are devoid of many disadvantages associated with the latter including high cost and the variable purity problems of phospholipids [80]. These vesicular carriers resemble liposomes in their morphology but contain unilayers of surfactants unlike phospholipid bilayers in liposomes, as indicated in Figure 6(d). In a comparative study with liposomes, Nasr et al. reported the better stability and efficacy of the developed niosomes than that of liposomes [67]. Solanki et al. also prepared ACE-loaded niosomes for the transdermal application and reported enhanced permeation of drug for an extended period of time employing rat skin. The anti-inflammatory efficacy was found to be superior to that of the plain gel evaluated on carrageenan-induced rat paw oedema [81]. Solanki et al. characterized and optimized ACE-loaded proniosomes (ready to use niosomes dry form) using central composite design and carried out stability studies, but* in vivo* pharmacodynamic and chemical stability studies were not furnished [82].

### 6.5. Organogel-Based Delivery

Organogels-based drug products are being preferred these days owing to their longer shelf-lives, better penetration ability, ease of preparation, thermoreversible nature, and ability to accommodate both hydrophilic and hydrophobic compounds. Organogels are the semisolid preparations which include gelator substance, nonpolar solvent, and a polar solvent [83]. They are generally envisioned as the reverse micelles embedded in the organogel, as shown in Figure 6(e) [43]. Shaikh et al. developed and evaluated lecithin organogels for the topical application attributes. The research findings report superior ACE skin delivery potential of the organogels* vis-à-vis *the hydrogels employing albino rat skin. The organogel was found to be more effective than the hydrogel in carrageenan-induced rat paw oedema and also the organogel was well-tolerated on rat abdominal skin as revealed by histopathological investigation [84, 85]. Kamble et al. developed and characterized the pluronic-lecithin-based organogels for the topical delivery of ACE and evaluated the release potential of the organogel employing dialysis membrane, though penetration enhancers were used. The anti-inflammatory efficacy was found to be superior to that of the standard marketed product assessed on carrageenan-induced rat paw oedema. The formulation was found to be nonirritant when tested on the skins of guinea pigs. They reported substantial gel stability on storage, though chemical stability remained undisclosed [86].

### 6.6. Liposphere-Based Delivery

Lipospheres are solid lipid particles in which the lipid particles ensure close contact with the SC and promise enhanced drug penetrating into the mucosa or skin. Lipospheres are composed of solid lipid core embedded with one monolayer of phospholipid molecules on the surface for stabilization and dermal penetration, as depicted in Figure 6(f) [87]. Nasr et al. formulated lipospheres for topical delivery of ACE and reported enhanced drug entrapping ability, high stability, and ability to sustain the anti-inflammatory action. The anti-inflammatory efficacy of prepared lipospheres was found to be superior to the marketed product when assessed on carrageenan-induced rat paw oedema. The lipospheres were able to maintain their physical attributes for 3-month storage at 2–8°C [87].

### 6.7. Lipid Carriers-Based Delivery

Nanostructure lipid carriers (NLC) are the new generation of lipid nanoparticles, consisting of a mixture of specially blended solid lipid (long chain) with liquid lipid (short chain), preferably in a ratio of 70 : 30 up to a ratio of 99.9 : 0.1, whereas the conventional solid lipid nanoparticles (SLNs) only employ solid lipids [36, 40, 44]. Unlike, SLNs, NLCs can have fluidic core so as to dissolve more of drug, as shown in Figures 6(g) and 6(h). Patel et al. formulated NLC-based topical gel of ACE and observed that the onset of action was faster and duration was sustained with the NLC-gel* vis-à-vis *the marketed product. The developed formulations were found to be nonirritant on the rat skin and, hence, inferred compliance on topical application [88].

Chawla and Saraf systematically developed SLNs of ACE and successfully incorporated these nanocarriers in carbopol hydrogel. They reported sustained release behavior of the developed system; however, the findings are related to the rheological attributes and release characteristics from nanocolloid-based hydrogels [89].

## 7. Conclusions

Due to advent in the patient-centric approaches aimed at the safety and efficacy enhancement of drugs, nanocolloidal drug delivery vehicles are frequently employed these days. Owing to the advantageous features, over 500 nanotechnology-based drug products have already been catalogued globally and the number is escalating in an exponential manner. The projected market of these nanosized drug products for 2015 is around $3.1 trillion. The fundamental interest of such carriers lies in making the existing drugs more safe, effective, and patient compliant. The studies enlisted suggest the importance of these systems in the enhanced skin penetration and accumulation of ACE along with improved patient compliance. The unique ability of these carriers and the interactions with the skin components are the possible reason for the better cutaneous transport of drugs, though liposomes are less explored in case of topical delivery of ACE.

Although substantial work has been done with respect to the preclinical evaluation of these carriers, still studies on the clinical evaluation of ACE-loaded novel carriers are almost missing. However, out of a few isolated attempts for clinical applications, the availability of ACE by topical route epitomizes the initiation of the progress. Holistic efforts from all domains including the pharmacoeconomic, formulatory, material science and preclinical and clinical stakeholders are desired to ensure the availability of such promising products to the real stakeholders, that is, the patients.

## Figures and Tables

**Table 1:** Advantages of topical delivery of ACE.

(i) Avoidance of hepatic first-pass metabolism	
(ii) Accessibility to the site of action	
(iii) Prevention of naive cells from toxic drugs conc.	
(iv) Discontinuation on desire	
(v) Drug delivery at controlled rate	
(vi) Fixed plasma drug levels (transdermal delivery)	
(vii) Economic benefits	
(viii) Patient compliance	

**Table 2:** Advantages of NDDS.

(i) Availability of versatile carriers	
(ii) Protection to drug molecules	
(iii) Biocompatible	
(iv) Interaction with skin components	
(v) Loading of variety of drugs	
(vi) Modification in physiochemical properties	
(vii) Intact penetration	
(viii) Passive targeting	

**Table 3:** Various novel carriers employed till date for the topical delivery of aceclofenac.

S. Number	Carrier system	Advantages/results obtained
1.	Liposome-mediated delivery [87]	Sustained anti-inflammatory activity
2.	Ethosomes-mediated delivery [69–72]	Enhancement in Skin Permeation, Improvement in Anti-Inflammatory Efficacy
3.	Microemulsion and nanoemulsion-mediated delivery [74–77]	Biocompatible, enhanced skin permeability, andefficacy
4.	Niosome-based delivery [81, 82, 87]	Enhanced permeability, efficacy, and* s*tability *vis-à-vis * liposomes
5.	Organogel-based delivery [83, 84, 86]	Superior efficacy andstability, nonirritant
6.	Liposphere-based delivery [87]	Enhanced stability, permeability, drug entrapment, andefficacy
7.	Nanostructured lipid carriers- (NLC-) based delivery [86]	Fast onset of action, sustained duration of action, nonirritant *vis-à-vis* the marketed products
8.	Solid lipid nanoparticles-mediated delivery [89]	Sustained drug release, better rheology
